# A rare positively charged nicotinic acid di­sulfide: 2,2′-di­thio­dinicotinic acid hydro­chloride monohydrate

**DOI:** 10.1107/S2056989018006916

**Published:** 2018-05-18

**Authors:** Alexander T. Chemey, Chad M. McGuire, Thomas E. Albrecht-Schmitt

**Affiliations:** a Department of Chemistry and Biochemistry, Florida State University, 95 Chieftan Way Tallahassee, FL 32306, USA

**Keywords:** crystal structure, di­sulfide bonds, hydrate, nicotinic acid, mercaptonicotinic acid, pyridinium

## Abstract

The title compound {systematic name: 3-carb­oxy-2-[2-(3-carb­oxy­pyridin-2-yl)disulfan-1-yl)]pyridin-1-ium chloride monohydrate}, C_12_H_9_N_2_O_4_S_2_^+^·Cl^−^·H_2_O, crystallizes in the triclinic space group *P* . A pair of 2-mercaptonicotinic acid moieties is connected by a 2,2′-di­sulfide bond with a dihedral angle of 78.79 (3)°. One of the N atom is protonated, as are both carboxyl­ate groups, resulting in an overall +1 charge on the dimer. The structure comprises a zigzagging layer of the dimerized di­thio­dinicotinic acid rings, with charge-balancing chloride ions and water mol­ecules between the layers. Hydrogen bonding between the chloride and water sites with the dimer appears to hold the structure together. Nearest neighbor nicotinic acid rings are offset when viewed down the *a* axis, suggesting no added stability from ring stacking. The asymmetric unit corresponds to the empirical formula of the compound, and it packs with two formula units per unit cell.

## Abstract 2

An unexpected product was obtained from slow evaporation in acidic media, as a protonated nitro­gen is combined with a di­sulfide bond between a pair of 2-mercaptonicotinic acid moieties.

## Chemical context   

2-Mercaptonicotinic acid (2-mnaH) is commonly used as a multi-dentate ligand. It is a flexible hard/soft ligand, capable of binding *via* a carboxyl­ate oxygen and sulfur, or nitro­gen and sulfur on a single metal site. This flexibility has been exploited in a variety of reactions with hard and soft metals to produce diverse structures. Mercaptonicotinic acid complexes with silver(I) and gold(I) have proven anti-microbial properties (Nomiya *et al.*, 2000 ▸), while transition metal–mna clusters have provided fertile ground for luminescence studies (Sun *et al.*, 2011 ▸). In some extraordinary cases, all three potential binding sites of 2-mna coordinate to metal centers (Humphrey *et al.*, 2006 ▸). 2-mnaH tends to form di­sulfide bonds with itself in neutral solutions, and these dimers have provided inter­esting coordination modes with the lanthanides (Li *et al.*, 2008 ▸; Wang *et al.*, 2011 ▸), as have related 2,2′-di­thio­disalicylic acid compounds (Zhong *et al.*, 2014 ▸). Di­sulfide formation is typically inhibited by the use of acidic solution, and these non-dimerized species often feature protonated nitro­gens, such as in HAu(mnaH)_2_ (Nomiya *et al.*, 2000 ▸). It is therefore quite unusual to have a di­sulfide bond form from a solution acidic enough to make a nicotinium species.

## Structural commentary   

**Figure 1:**
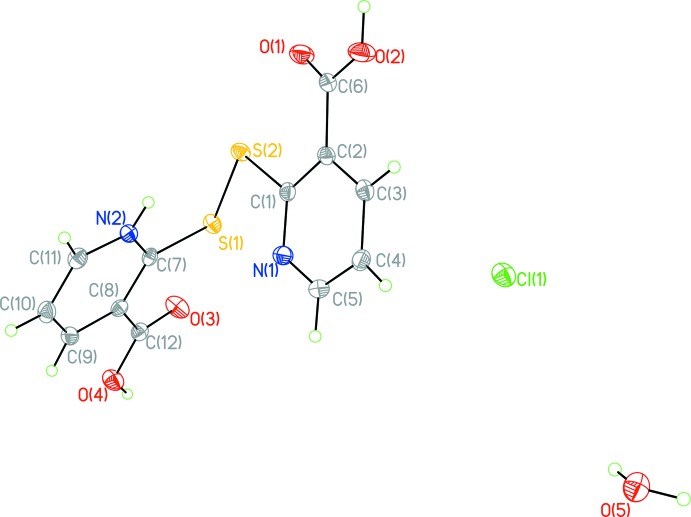
Displacement ellipsoid plot (50% probability level) of H(2-mnaH)_2_Cl·H_2_O.

The title compound (Fig. 1 ▸), referred to as H(2-mnaH)_2_Cl·H_2_O), crystallizes in the triclinic space group *P* 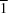. A pair of 2-mnaH moieties is connected by a 2.0491 (9) Å di­sulfide bond with a dihedral angle of 78.79 (3)°. The structure features a zigzagging layer structure. The presence of a chloride in the structure mandates an overall positive charge on the dimerized species, as there is no evidence of the co-crystallized water being a hydro­nium ion. One nitro­gen is protonated and the nicotinate ring which incorporates that charge is assigned the descriptor of α moiety. Both carb­oxy­late groups are protonated.
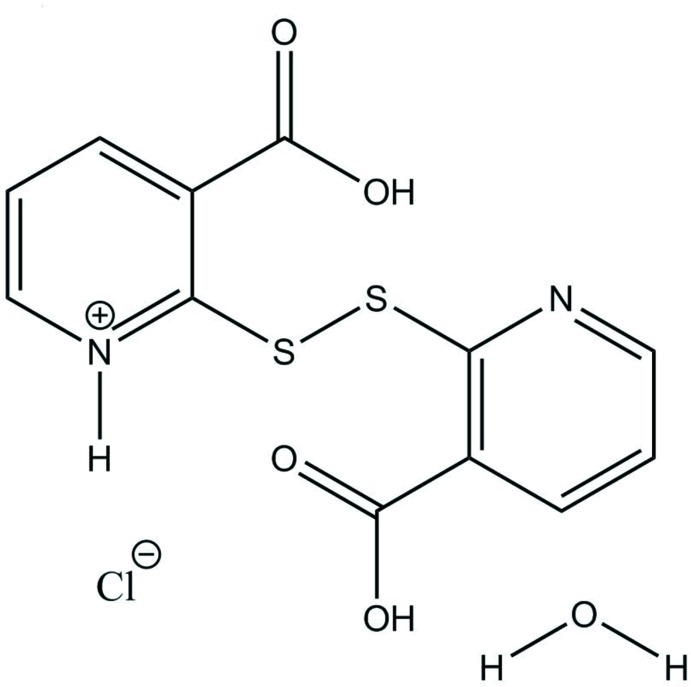


## Supra­molecular features   

**Figure 2:**
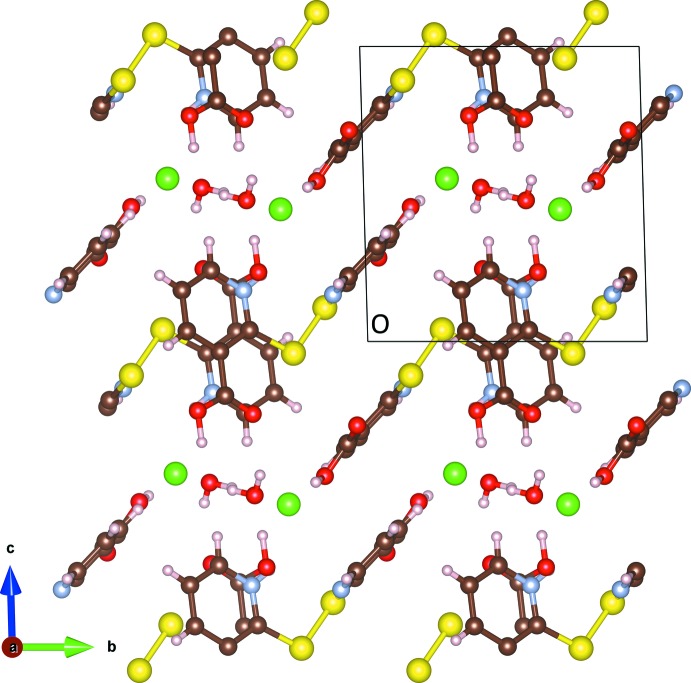
The packing of H(2-mnaH)_2_Cl·H_2_O, as viewed down the *a* axis. Note the zigzag shape of the layers, and the offset configuration of the nicotinic acid rings. Color code: yellow, sulfur; brown, carbon; pale blue, nitro­gen; green, chlorine; pale pink, hydrogen.

**Figure 3:**
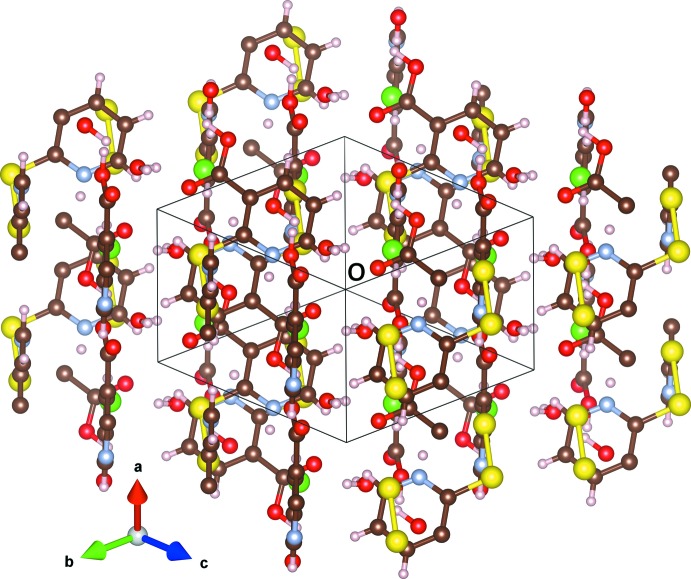
The view down [111], illustrating the inter-layer chloride and water sites which stitch the cationic dimers together.

Nicotinic acid rings are offset when viewed down the *a* axis with distances of 3.822 (3) Å between nearest neighbor ring-bound atoms. This is illustrated in Fig. 2 ▸. The H(2-mnaH)_2_^+^ layers stack along the *c* axis. Between the layers are the water mol­ecules and chloride anions, which hold the layers together through hydrogen bonding (Table 1 ▸). These inter-layer sites are visible when viewed along [111], as seen in Fig. 3 ▸. The acidic hydrogen on the α carboxyl­ate (H4) is 1.52 (3) Å from the co-crystallized water oxygen atom (O5), apparently pointing at a lone pair, with a total O4—O5 distance of only 2.5164 (19) Å. This close proximity is likely the cause of the long O4—H4 distance of 1.01 (3) Å, as the hydrogen is significantly attracted to the lone pair of O5. The β moiety acidic hydrogen (H3) is pointing almost directly at the inter-layer chloride. The co-crystallized water has one hydrogen pointing towards the chloride (H2), and one hydrogen (H1) pointing approximately towards the doubly-bonded oxygen (O3) in the α moiety. The protonated nitro­gen points into space between the α-connected sulfur and the chloride ion. Hydrogen bonding with the chloride anions and co-crystallized water mol­ecules appears to dictate the staggered layered structure, but the potential for π–π inter­actions between parallel rings cannot be excluded.

## Database survey   

The di­sulfide dihedral angle of H(2-mnaH)_2_Cl·H_2_O is comparable to the dihedral angles observed in 2,2′-disulfan­yl­disalicylic acid of 74.9 (8) and 77.7 (6)° (Humphrey & Wood, 2003 ▸), but notably smaller than the values of 88.70 (6) and 89.22 (6)° from the structure published by Rowland *et al.* (2011 ▸). In the absence of a bulky metal cation, Yehye *et al.* (2009 ▸) measured a 2,2′-di­thio­dinicotinate dihedral angle of 89.2 (2)°. Two different dimerized 2-mna dysprosium compounds featured dihedral angles of 84.85 (6) (Wang *et al.*, 2011 ▸), 88.77 (4) and 0.00° (Li *et al.*, 2008 ▸). The second structure is an exciting example of a lanthanide-di­thio­dinicotinate with two separate dimer moieties and shockingly different dihedral angles. Slightly different structures of 2,2′-disulfan­yldinicotinic acid have been produced as the monohydrate (Corban *et al.*, 2012 ▸; Zhu *et al.*, 2002 ▸); these structures have dihedral angles of 87.43 (3) and 87.33 (4)°, respectively.

*In* situ production of the dimer has been exploited in several syntheses to make a harder ligand with binding predominantly through the carb­oxy­lic oxygens (Singh *et al.*, 2012 ▸; Li *et al.*, 2008 ▸). Similar reactivity of the sulfur site has been accomplished with 2-thio­salicylic acid to produce a simple dimer (Humphrey & Wood, 2003 ▸), while a broader study explored the products obtained with heating and variable pH (Rowland *et al.*, 2011 ▸). In that study, acidic environments prevented di­sulfide formation at pH even as high as 4.4, a strong contrast to the species here. Four structures have been reported to the CSD (Version 1.18; Groom *et al.*, 2016 ▸) with a protonated nitro­gen on 2-mnaH, none of which featured di­sulfides. One structure featured sulfur sites binding to gold(I) (Nomiya *et al.*, 2000 ▸), while another was bonded through the sulfur and an oxygen site to zinc(II) (Casarin *et al.*, 2010 ▸). The other two structures were zwitterionic monomers featuring overall neutral or negative charges on the nicotinic acid moiety (Smith & Sagatys, 2003 ▸; Kouroulis *et al.*, 2009 ▸). There is no positively charged 2-mnaH moiety currently listed in the CSD. There is also no nitro­gen-protonated di­sulfide nicotinic species in the CSD.

In contrast to work with 2,2′-disulfanyldisalicylic acid (Humphrey & Wood, 2003 ▸; Rowland *et al.*, 2011 ▸), hydrogen bonding directly between carb­oxy­lic acid groups does not appear to play a large role. Although it is not possible to exclude the possibility that the pyridinium hydrogen atom is engaged in hydrogen bonding, as in studies of ammonium 2-mercapto­pyridine-3-carboxyl­ate hydrate (Smith & Sagatys, 2003 ▸), the location of the pyridinium in space makes this less likely.

## Synthesis and crystallization   

H(2-mnaH)_2_Cl·H_2_O was grown from 23.5 mg of 2-mnaH (90+%, Alfa Aesar) with 30 mmol of 1 mol/L Ho(NO_3_)_3_ in 1 mL of a mixture of methanol and ethanol with three drops of concentrated HCl. The composition of the alcohol mixture was varied systematically from pure ethanol to pure methanol in 10% increments, and crystals were examined in an optical microscope to determine size and quality. The largest and least-occluded crystals (∼0.5 × 0.2 × 0.2 mm^3^ prior to cutting) were grown from a 70% ethanol/30% methanol mixture, though crystals that were smaller and diffracted weakly were grown from more methanol-rich solutions. Both methanol-rich and ethanol-rich solutions produced the same structures. The components were heated in a 7 mL vial with the lid off for twenty minutes at 325 K, though crystals similar to those grown from methanol-rich media were grown without the heating step. Comparable reactions with 1 mol L^−1^ HoCl_3_ and 1 mol L^−1^ PrCl_3_ yielded no visible crystals, while reactions with 1 mol L^−1^ Pr(NO_3_)_3_ produced visually similar crystals that diffracted poorly. Reactions which proceded without a lanthanide nitrate did not produce single crystals, as did all reactions without acid. It is uncertain at this time whether a metal or nitrate is necessary for these crystals to grow.

Lanthanide chlorides and nitrates were produced from heating Ho_2_O_3_ (Aldrich, 99.9%) and Pr_6_O_11_ (Aldrich, 99.9%) in the presence of hydro­chloric or nitric acid to dryness, to produce Ho(NO_3_)_3_·5H_2_O, HoCl_3_·6H_2_O, PrCl_3_·7H2O, and Pr(NO_3_)_3_·6H_2_O. Stock solutions were produced after weighing out a known amount of the hydrated species and dissolving in an appropriate amount of water for the desired concentration. Reproduction experiments determined the pH with a Fisher Scientific AB15 pH Meter calibrated with pH 4, 7, and 10 buffers. The pH before acidification of a 9.2 mg/1.185 mL (0.050 mol L^−1^) 2-mnaH solution was 4.30, and the pH after acidification with 0.150 mL of 12.1 mol L^−1^ HCl was determined to be −0.44.

The crystals grew as large blocky yellow octa­hedra on the base of the vial. A large crystal was cut down to an appropriate size. Single crystals were isolated from Krytox oil with CryoLoops, then optically aligned on a Bruker D8 Quest X-ray diffractometer using a digital camera. Initial intensity measurements were performed using a I*μ*S X-ray source, a 30 W microfocused sealed tube (Mo K*α*, λ = 0.71073 Å) with high-brilliance and high-performance focusing Qu­azar multilayer optics. Standard *APEX3* software was used for determination of the unit cells and data collection control. The intensities of reflections of a sphere were collected by a combination of four sets of exposures (frames). Each set had a different *φ* angle for the crystal and each exposure covered a range of 0.5^o^ in *ω*. A total of 1464 frames were collected with an exposure time per frame of 20 s. *SAINT* software was used for data integration including Lorentz and polarization corrections. A semi-empirical absorption correction was applied using the program SCALE (*SADABS*).

## Refinement   

Crystal data, data collection and structure refinement details are summarized in Table 2 ▸.

Prospective peaks at appropriate distances from the 4, 5, and 6 carbon positions were observed on both rings and allowed to refine independently of their carbons. Hydrogen atoms were also observed as prospective peaks at distances of approximately 1 Å from a carb­oxy­lic oxygen for both moieties, and 0.8-0.9 Å from the co-crystallized water oxygen. The command HADD in *SHELXP* suggested potential hydrogen atoms at reasonable distances from nitro­gen sites, but refinement of the structure led to one of the atoms detaching from the structure. Free refinement of occupancy for the remaining nitro­genic hydrogen resulted in a site occupancy factor of over 80%, so occupancy was therefore fixed to 1. Bond valence sum calculations of the structure excluding carb­oxy­lic hydrogens found that two of the four oxygens were particularly distant from ideal valency; these oxygens were closest to the unaccounted peaks previously identified, and the hydrogens were therefore assigned despite their greater-than-expected distance. Attempts to refine hydrogens on the co-crystallized water and carboxyl­ate groups with AFIX restraints by *SHELXL* failed to yield reasonable results. Free refinement of occupancy for the acidic hydrogens resulted in non-physical values, so site occupancy was fixed at 1. These results led to an overall dimer charge of +1, and a balanced charge state with the inter­layer chloride.

*PLATON* (Spek, 2009 ▸) was used to check for unresolved solvent electron density, additional symmetry, and twinning.

## Supplementary Material

Crystal structure: contains datablock(s) I. DOI: 10.1107/S2056989018006916/yk2114sup1.cif

Structure factors: contains datablock(s) I. DOI: 10.1107/S2056989018006916/yk2114Isup2.hkl

Supporting information file. DOI: 10.1107/S2056989018006916/yk2114Isup3.cml

CCDC reference: 1841479

Additional supporting information:  crystallographic information; 3D view; checkCIF report

## Figures and Tables

**Table 1:** Hydrogen-bond geometry (Å, °)

*D*—H⋯*A*	*D*—H	H⋯*A*	*D*⋯*A*	*D*—H⋯*A*
N2—H2*A*⋯Cl1^i^	0.84 (2)	2.41 (2)	3.1228 (15)	143 (2)
C10—H10⋯O1^ii^	0.91 (2)	2.53 (2)	3.405 (2)	162.0 (18)
C11—H11⋯Cl1^iii^	0.94 (2)	2.74 (2)	3.4776 (18)	135.7 (15)
C4—H4*A*⋯O1^iv^	0.90 (2)	2.63 (2)	3.259 (2)	128.5 (17)
C4—H4*A*⋯Cl1	0.90 (2)	2.89 (2)	3.6340 (19)	141.1 (17)
O4—H4⋯O5^v^	1.01 (3)	1.52 (3)	2.5164 (19)	169 (3)
O2—H3⋯Cl1^vi^	0.91 (3)	2.12 (3)	3.0197 (15)	170 (3)
O5—H1⋯O3^vii^	0.85 (3)	2.10 (3)	2.8709 (19)	151 (3)
O5—H2⋯Cl1^viii^	0.88 (3)	2.20 (3)	3.0848 (16)	177 (3)

Symmetry codes: (i) 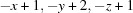; (ii) 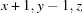; (iii) 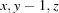; (iv) 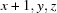; (v) 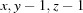; (vi) 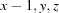; (vii) 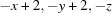; (viii) 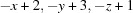.

**Table 2:** Experimental details

Crystal data	
Chemical formula	C_12_H_9_N_2_O_4_S_2_^+^·Cl^−^·H_2_O
*M*_r_	362.80
Crystal system, space group	Triclinic, *P* 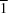
Temperature (K)	120
*a*, *b*, *c* (Å)	7.9906 (12), 9.7081 (14), 10.2704 (15)
α, β, γ (°)	86.727 (3), 73.088 (3), 73.538 (3)
*V* (Å^3^)	730.73 (19)
*Z*	2
Radiation type	Mo *K*α
μ (mm^−1^)	0.57
Crystal size (mm)	0.05 × 0.05 × 0.03

Data collection	
Diffractometer	Bruker D8 Quest
Absorption correction	Multi-scan (*SADABS*; Bruker, 2015 ▸)
*T*_min_, *T*_max_	0.688, 0.747
No. of measured, independent and observed [*I* > 2σ(*I*)] reflections	11750, 3483, 2974
*R*_int_	0.031
(sin θ/λ)_max_ (Å^−1^)	0.658

Refinement	
*R*[*F* ^2^ > 2σ(*F* ^2^)], *wR*(*F* ^2^), *S*	0.032, 0.075, 1.06
No. of reflections	3483
No. of parameters	243
H-atom treatment	All H-atom parameters refined
Δρ_max_, Δρ_min_ (e Å^−3^)	0.34, −0.29

Computer programs: *APEX3* and *SAINT* (Bruker, 2015 ▸), *SHELXS2014* (Sheldrick, 2008 ▸), *SHELXL2014* and *SHELXP2014* (Sheldrick, 2015 ▸), *VESTA* (Momma & Izumi, 2011 ▸) and *publCIF* (Westrip, 2010 ▸).

## supplementary crystallographic information

### Crystal data

**Table:**

C_12_H_9_N_2_O_4_S_2_^+^·Cl^−^·H_2_O	*Z* = 2
*M**_r_* = 362.80	*F*(000) = 372
Triclinic, *P*1	*D*_x_ = 1.649 Mg m^−^^3^
*a* = 7.9906 (12) Å	Mo *K*α radiation, λ = 0.71073 Å
*b* = 9.7081 (14) Å	Cell parameters from 2974 reflections
*c* = 10.2704 (15) Å	θ = 2.8–27.9°
α = 86.727 (3)°	µ = 0.57 mm^−^^1^
β = 73.088 (3)°	*T* = 120 K
γ = 73.538 (3)°	Truncated column, yellow
*V* = 730.73 (19) Å^3^	0.05 × 0.05 × 0.03 mm

### Data collection

**Table:**

Bruker D8 Quest diffractometer	2974 reflections with *I* > 2σ(*I*)
Radiation source: Iµs microfocused	*R*_int_ = 0.031
0.5° wide /w exposures scans	θ_max_ = 27.9°, θ_min_ = 2.8°
Absorption correction: multi-scan (SADABS; Bruker, 2015)	*h* = −10→10
*T*_min_ = 0.688, *T*_max_ = 0.747	*k* = −12→12
11750 measured reflections	*l* = −13→13
3483 independent reflections	

### Refinement

**Table:**

Refinement on *F*^2^	0 restraints
Least-squares matrix: full	Hydrogen site location: difference Fourier map
*R*[*F*^2^ > 2σ(*F*^2^)] = 0.032	All H-atom parameters refined
*wR*(*F*^2^) = 0.075	*w* = 1/[σ^2^(*F*_o_^2^) + (0.028*P*)^2^ + 0.4761*P*] where *P* = (*F*_o_^2^ + 2*F*_c_^2^)/3
*S* = 1.06	(Δ/σ)_max_ = 0.001
3483 reflections	Δρ_max_ = 0.34 e Å^−^^3^
243 parameters	Δρ_min_ = −0.28 e Å^−^^3^

### Special details

**Table:**

Geometry. All esds (except the esd in the dihedral angle between two l.s. planes) are estimated using the full covariance matrix. The cell esds are taken into account individually in the estimation of esds in distances, angles and torsion angles; correlations between esds in cell parameters are only used when they are defined by crystal symmetry. An approximate (isotropic) treatment of cell esds is used for estimating esds involving l.s. planes.

### Fractional atomic coordinates and isotropic or equivalent isotropic displacement parameters (Å^2^)

**Table:**

	*x*	*y*	*z*	*U*_iso_*/*U*_eq_	
S1	0.55285 (6)	0.73261 (4)	−0.03978 (4)	0.01593 (10)	
S2	0.34334 (6)	0.84956 (5)	0.11499 (4)	0.01655 (10)	
O4	1.03798 (18)	0.38461 (14)	−0.23492 (13)	0.0231 (3)	
N1	0.6456 (2)	0.88005 (16)	0.15917 (15)	0.0183 (3)	
O3	0.81335 (18)	0.58125 (14)	−0.24563 (12)	0.0241 (3)	
O2	0.09614 (18)	1.16658 (15)	0.45610 (14)	0.0249 (3)	
C12	0.8861 (2)	0.48353 (18)	−0.18308 (17)	0.0160 (3)	
N2	0.5831 (2)	0.55966 (15)	0.17321 (14)	0.0137 (3)	
H2A	0.495 (3)	0.625 (3)	0.219 (2)	0.033 (6)*	
O1	0.07437 (17)	1.04648 (14)	0.28410 (14)	0.0243 (3)	
C9	0.8823 (2)	0.35342 (18)	0.03531 (18)	0.0164 (3)	
H9A	0.987 (3)	0.286 (2)	−0.013 (2)	0.015 (5)*	
C10	0.8052 (2)	0.33966 (19)	0.17376 (18)	0.0181 (4)	
H10	0.854 (3)	0.263 (2)	0.220 (2)	0.022 (5)*	
C11	0.6531 (2)	0.44517 (19)	0.24083 (18)	0.0169 (3)	
H11	0.596 (3)	0.443 (2)	0.334 (2)	0.016 (5)*	
C5	0.7374 (2)	0.93776 (19)	0.22147 (19)	0.0196 (4)	
H5A	0.869 (3)	0.901 (2)	0.192 (2)	0.021 (5)*	
C7	0.6516 (2)	0.57744 (17)	0.03867 (16)	0.0129 (3)	
C1	0.4649 (2)	0.92968 (17)	0.19882 (17)	0.0148 (3)	
C4	0.6562 (2)	1.04237 (18)	0.32312 (18)	0.0174 (3)	
H4A	0.723 (3)	1.075 (2)	0.364 (2)	0.025 (6)*	
C3	0.4674 (2)	1.09120 (18)	0.36465 (17)	0.0160 (3)	
H3A	0.408 (3)	1.162 (2)	0.430 (2)	0.014 (5)*	
C2	0.3676 (2)	1.03471 (17)	0.30221 (17)	0.0148 (3)	
C8	0.8054 (2)	0.46990 (17)	−0.03376 (16)	0.0139 (3)	
C6	0.1649 (2)	1.08231 (18)	0.34541 (18)	0.0168 (3)	
H4	1.093 (4)	0.396 (3)	−0.336 (3)	0.058 (8)*	
H3	−0.028 (4)	1.198 (3)	0.478 (3)	0.059 (9)*	
O5	1.20128 (19)	1.41990 (16)	0.52156 (14)	0.0237 (3)	
H1	1.173 (4)	1.400 (3)	0.453 (3)	0.064 (9)*	
H2	1.232 (4)	1.501 (3)	0.504 (3)	0.062 (9)*	
Cl1	0.68897 (6)	1.29911 (4)	0.55204 (4)	0.01899 (11)	

### Atomic displacement parameters (Å^2^)

**Table:**

	*U*^11^	*U*^22^	*U*^33^	*U*^12^	*U*^13^	*U*^23^
S1	0.0192 (2)	0.0151 (2)	0.01288 (19)	−0.00254 (16)	−0.00613 (16)	0.00176 (15)
S2	0.0152 (2)	0.0167 (2)	0.0180 (2)	−0.00215 (16)	−0.00707 (16)	−0.00083 (16)
O4	0.0219 (7)	0.0223 (7)	0.0173 (6)	−0.0006 (5)	0.0010 (5)	−0.0002 (5)
N1	0.0158 (7)	0.0178 (7)	0.0211 (7)	−0.0038 (6)	−0.0052 (6)	−0.0020 (6)
O3	0.0269 (7)	0.0260 (7)	0.0130 (6)	0.0015 (6)	−0.0047 (5)	0.0003 (5)
O2	0.0154 (6)	0.0293 (7)	0.0256 (7)	−0.0027 (6)	−0.0009 (5)	−0.0087 (6)
C12	0.0173 (8)	0.0159 (8)	0.0151 (8)	−0.0060 (7)	−0.0031 (6)	−0.0028 (6)
N2	0.0143 (7)	0.0137 (7)	0.0127 (7)	−0.0034 (6)	−0.0036 (5)	−0.0006 (5)
O1	0.0157 (6)	0.0252 (7)	0.0315 (7)	−0.0023 (5)	−0.0078 (5)	−0.0064 (6)
C9	0.0159 (8)	0.0142 (8)	0.0195 (8)	−0.0039 (7)	−0.0054 (7)	−0.0012 (7)
C10	0.0217 (9)	0.0166 (8)	0.0192 (8)	−0.0072 (7)	−0.0098 (7)	0.0054 (7)
C11	0.0192 (9)	0.0192 (9)	0.0143 (8)	−0.0078 (7)	−0.0059 (7)	0.0037 (7)
C5	0.0146 (9)	0.0184 (9)	0.0257 (9)	−0.0045 (7)	−0.0055 (7)	0.0001 (7)
C7	0.0156 (8)	0.0134 (8)	0.0124 (7)	−0.0069 (6)	−0.0055 (6)	0.0008 (6)
C1	0.0163 (8)	0.0121 (8)	0.0167 (8)	−0.0049 (6)	−0.0054 (6)	0.0025 (6)
C4	0.0184 (9)	0.0156 (8)	0.0204 (9)	−0.0073 (7)	−0.0068 (7)	0.0019 (7)
C3	0.0187 (8)	0.0129 (8)	0.0153 (8)	−0.0033 (7)	−0.0044 (7)	0.0013 (6)
C2	0.0146 (8)	0.0129 (8)	0.0159 (8)	−0.0028 (6)	−0.0042 (6)	0.0035 (6)
C8	0.0144 (8)	0.0155 (8)	0.0137 (8)	−0.0066 (6)	−0.0042 (6)	−0.0008 (6)
C6	0.0148 (8)	0.0122 (8)	0.0214 (9)	−0.0027 (6)	−0.0036 (7)	0.0019 (6)
O5	0.0272 (7)	0.0252 (7)	0.0181 (7)	−0.0094 (6)	−0.0031 (5)	−0.0020 (6)
Cl1	0.0198 (2)	0.0182 (2)	0.0150 (2)	−0.00300 (16)	−0.00111 (16)	0.00010 (15)

### Geometric parameters (Å, º)

**Table:**

S1—C7	1.7602 (17)	C9—C10	1.391 (2)
S1—S2	2.0491 (6)	C9—H9A	0.93 (2)
S2—C1	1.8113 (17)	C10—C11	1.374 (3)
O4—C12	1.304 (2)	C10—H10	0.91 (2)
O4—H4	1.01 (3)	C11—H11	0.94 (2)
N1—C1	1.330 (2)	C5—C4	1.377 (3)
N1—C5	1.345 (2)	C5—H5A	0.97 (2)
O3—C12	1.219 (2)	C7—C8	1.408 (2)
O2—C6	1.324 (2)	C1—C2	1.402 (2)
O2—H3	0.91 (3)	C4—C3	1.388 (2)
C12—C8	1.495 (2)	C4—H4A	0.90 (2)
N2—C11	1.348 (2)	C3—C2	1.390 (2)
N2—C7	1.351 (2)	C3—H3A	0.912 (19)
N2—H2A	0.84 (2)	C2—C6	1.489 (2)
O1—C6	1.214 (2)	O5—H1	0.85 (3)
C9—C8	1.388 (2)	O5—H2	0.88 (3)
			
C7—S1—S2	104.35 (6)	N2—C7—C8	117.59 (15)
C1—S2—S1	101.27 (6)	N2—C7—S1	120.27 (12)
C12—O4—H4	113.0 (16)	C8—C7—S1	122.13 (12)
C1—N1—C5	116.90 (15)	N1—C1—C2	123.81 (15)
C6—O2—H3	111.6 (18)	N1—C1—S2	116.31 (13)
O3—C12—O4	125.39 (16)	C2—C1—S2	119.85 (13)
O3—C12—C8	120.99 (15)	C5—C4—C3	118.05 (17)
O4—C12—C8	113.61 (15)	C5—C4—H4A	121.2 (14)
C11—N2—C7	123.70 (15)	C3—C4—H4A	120.7 (14)
C11—N2—H2A	117.0 (16)	C4—C3—C2	119.45 (16)
C7—N2—H2A	119.2 (16)	C4—C3—H3A	121.1 (12)
C8—C9—C10	120.68 (16)	C2—C3—H3A	119.4 (12)
C8—C9—H9A	118.4 (12)	C3—C2—C1	117.55 (15)
C10—C9—H9A	120.9 (12)	C3—C2—C6	121.11 (15)
C11—C10—C9	118.45 (16)	C1—C2—C6	121.33 (15)
C11—C10—H10	120.2 (13)	C9—C8—C7	119.36 (15)
C9—C10—H10	121.4 (13)	C9—C8—C12	120.77 (15)
N2—C11—C10	120.19 (16)	C7—C8—C12	119.86 (15)
N2—C11—H11	117.2 (12)	O1—C6—O2	124.40 (16)
C10—C11—H11	122.6 (12)	O1—C6—C2	122.64 (16)
N1—C5—C4	124.23 (16)	O2—C6—C2	112.96 (15)
N1—C5—H5A	116.2 (12)	H1—O5—H2	107 (3)
C4—C5—H5A	119.6 (12)		

### Hydrogen-bond geometry (Å, º)

**Table:**

*D*—H···*A*	*D*—H	H···*A*	*D*···*A*	*D*—H···*A*
N2—H2*A*···Cl1^i^	0.84 (2)	2.41 (2)	3.1228 (15)	143 (2)
C10—H10···O1^ii^	0.91 (2)	2.53 (2)	3.405 (2)	162.0 (18)
C11—H11···Cl1^iii^	0.94 (2)	2.74 (2)	3.4776 (18)	135.7 (15)
C4—H4*A*···O1^iv^	0.90 (2)	2.63 (2)	3.259 (2)	128.5 (17)
C4—H4*A*···Cl1	0.90 (2)	2.89 (2)	3.6340 (19)	141.1 (17)
O4—H4···O5^v^	1.01 (3)	1.52 (3)	2.5164 (19)	169 (3)
O2—H3···Cl1^vi^	0.91 (3)	2.12 (3)	3.0197 (15)	170 (3)
O5—H1···O3^vii^	0.85 (3)	2.10 (3)	2.8709 (19)	151 (3)
O5—H2···Cl1^viii^	0.88 (3)	2.20 (3)	3.0848 (16)	177 (3)

Symmetry codes: (i) −*x*+1, −*y*+2, −*z*+1; (ii) *x*+1, *y*−1, *z*; (iii) *x*, *y*−1, *z*; (iv) *x*+1, *y*, *z*; (v) *x*, *y*−1, *z*−1; (vi) *x*−1, *y*, *z*; (vii) −*x*+2, −*y*+2, −*z*; (viii) −*x*+2, −*y*+3, −*z*+1.
